# Driving digitisation of the workforce. Junior doctor's digital devices rollout at South London and Maudsley NHS Foundation Trust: an early review of uptake, usage and effects

**DOI:** 10.1192/bjo.2021.209

**Published:** 2021-06-18

**Authors:** Tharun Zacharia

**Affiliations:** South London and Maudsley NHS Foundation Trust, IoPPN King's College London

## Abstract

**Aims:**

A Quality Improvement Project (QIP) was completed at South London and Maudsley NHS Foundation Trust (SLaM) to provide access of Trust laptops and smartphones (digital devices) to training doctors.

The aim of the study was to assess the views of trainees before and after the rollout of digital devices. Also, to assess barriers of device uptake/usage.

**Method:**

Trainee doctors were surveyed before mass release of digital devices. Trainees were surveyed again 2 months later, providing ample opportunity to request a device.

Correlated survey questions asked about training grade, expectation to request a digital device (and subsequent completed request), expected usage cases (and actual usage cases), expected benefits (and actual benefits), perceived importance of access to each device (before and after access) and barriers for device uptake. Also inquired were general comments about the project and actual lead time on device access.

**Result:**

110, mixed training grade, doctors participated in both surveys combined. There was a high demand for digital devices. Laptop requests were highest, with greater clarity of potential usage cases noted for a laptop. Laptops were perceived to be the more important device to a trainee.

Common usage cases for laptops were clinical work (in and out of working hours). There was also high usage around educational and audit/QIP activities. Smartphones were used only for clinical work (in and out of working hours).

Having access to each device was most beneficial in saving clinician time. Other sizable benefits included improvements in communication with patients and other professionals, as well as benefits toward patient safety. The laptop specifically also improved educational access.

A larger proportion of trainees requested a mobile phone than were previously expecting to. New ways of working due to the pandemic were noted to be motivating factors toward usage of devices.

The biggest barrier to device uptake was the delay from request to acquisition (often over 4 weeks).

Numerous respondents used personal devices for Trust related activities. This was more prevalent with smartphones, than with laptops.

**Conclusion:**

Digital device rollout was a valued endeavour, with enhanced demand secondary to the pandemic. Primary usage and benefits supported clinical effectiveness. The primary access barrier was the delay from point of request to point of delivery. As SLaM, and the NHS as a whole, becomes increasingly digitised, this program is vital to allow the Trust to ensure the trainee workforce are digitally equipped to continue to deliver safe, effective and quality care.

